# Retraction Note: The assessment of the protective impact of spidroin extract against UV-A radiation damage by using earthworms (Aporrectodea caliginosa) as a robust human skin model via macroscopic and histological observations

**DOI:** 10.1007/s11356-024-33994-4

**Published:** 2024-06-17

**Authors:** Fatma El-Zahraa A. Abd El-Aziz, May S. Ismail, Ahmad El Askary, Attalla F. El-kott, Ahmed A. Tantawy

**Affiliations:** 1 Zoology Department, Faculty of Science, Assiut University, Assiut, Egypt; 2 Pharmaceutics Department, Faculty of Pharmacy, South Valley University, Qena, Egypt; 3 Department of Clinical Laboratory Sciences, College of Applied Medical Sciences, Taif University, P.O.Box 11099, Taif, 21944 Saudi Arabia; 4 Biology Department, Faculty of Science, King Khalid University, Abha, 61421 Saudi Arabia; 5 Zoology Department, College of Science, Damanhour University, Damanhour, 22511 Egypt; 6 Biotechnology Department, Faculty of Science, Zagazig University, Zagazig, Egypt

**Retraction Note: Environmental Science and Pollution Research (2022) 29:44906-44916**

10.1007/s11356-022-18861-4

The Editor-in-Chief has retracted this article. After publication, concerns were raised regarding highly similar images in Fig. 4e and f. The authors have stated that the images are different, but have not addressed the possibility of these images being serial sections from the same animal.

The authors have provided the full set of images from the experiments presented in Figs. 2-4. Further checks by the Publisher found additional similarities in the original images from different groups. The Editor-in-Chief therefore no longer has confidence in the presented data.

Ahmed A. Tantawy does not agree to this retraction. None of the other authors have responded to any correspondence from the publisher about this retraction.

